# The role of social capital in women’s sexual and reproductive health and rights in humanitarian settings: a systematic review of qualitative studies

**DOI:** 10.1186/s13031-021-00421-1

**Published:** 2021-11-24

**Authors:** Hannah Ireland, Nguyen Toan Tran, Angela Dawson

**Affiliations:** 1 Australian Centre for Public and Population Health Research, Faculty of Health, University of Technology Sydney, PO Box 123, Sydney, NSW 2007 Australia; 2 Faculty of Medicine, University of Geneva, Rue Michel-Servet 1, 1206 Geneva, Switzerland

**Keywords:** Sexual and reproductive health and rights, Social capital, Humanitarian, Social support, Conflict, Natural disaster, Systematic review, Qualitative

## Abstract

**Background:**

Social capital is an important social determinant of women’s sexual and reproductive health and rights. Little research has been conducted to understand the role of social capital in women’s sexual and reproductive health and how this can be harnessed to improve health in humanitarian settings. We synthesised the evidence to examine the nexus of women’s sexual and reproductive health and rights and social capital in humanitarian contexts.

**Methods:**

We undertook a systematic review of qualitative studies. The preferred reporting items for systematic review and meta-analysis guidelines were used to identify peer-reviewed, qualitative studies conducted in humanitarian settings published since 1999. We searched CINAHL, MEDLINE, ProQuest Health & Medicine, PubMed, Embase and Web of science core collection and assessed quality using the Critical Appraisal Skills Programme tool. We used a meta-ethnographic approach to synthesise and analyse the data.

**Findings:**

Of 6749 initially identified studies, we included 19 studies, of which 18 were in conflict-related humanitarian settings and one in a natural disaster setting. The analysis revealed that the main form of social capital available to women was bonding social capital or strong links between people within groups of similar characteristics. There was limited use of bridging social capital, consisting of weaker connections between people of approximately equal status and power but with different characteristics. The primary social capital mechanisms that played a role in women’s sexual and reproductive health and rights were social support, informal social control and collective action. Depending on the nature of the values, norms and traditions shared by network members, these social capital mechanisms had the potential to both facilitate and hinder positive health outcomes for women.

**Conclusions:**

These findings demonstrate the importance of understanding social capital in planning sexual and reproductive health responses in humanitarian settings. The analysis highlights the need to investigate social capital from an individual perspective to expose the intra-network dynamics that shape women’s experiences. Insights could help inform community-based preparedness and response programs aimed at improving the demand for and access to quality sexual and reproductive health services in humanitarian settings.

**Supplementary Information:**

The online version contains supplementary material available at 10.1186/s13031-021-00421-1.

## Background

In 2018, 136 million people needed humanitarian aid as a result of being affected by conflict, hazards, pandemics and displacement. Of these, 34 million were women of reproductive age and five million were pregnant [1]. Meeting the reproductive health needs of women and girls in these complex contexts requires the provision of and demand for quality services, and the recognition that sexual and reproductive rights—frequently violated in humanitarian settings—are human rights. These include, among others, the right to make autonomous decisions about one’s body, to engage in consensual sexual relations, to choose sexual partners, to choose whether to have children and if so how to space them, to have access to sexual and reproductive information, to terminate a pregnancy safely and to access sexual and reproductive healthcare free from coercion, discrimination and violence [2]. The minimum initial service package (MISP) for Sexual and Reproductive Health in Crises, developed for responding to reproductive health needs when a crisis occurs, reflects the integrated nature of sexual and reproductive health (SRH) and provides a concrete set of objectives in the areas of coordination, targeted clinical services and planning for the transition to comprehensive SRH services [3].

Social determinants impact women’s sexual and reproductive health and rights (SRHR) in a myriad of ways. A key social determinant of health and wellbeing is social capital. In the field of public health research social capital has been approached from two different perspectives. A social cohesion approach, developed first by Coleman [4] and built on by Putnam [5], sees social capital as the resources (e.g., trust, norms, exercise of sanctions) which are available, collectively, to members of a social group [6]. This is in contrast to a social network approach, first conceptualised by Bourdieu [7] and later developed by Lin [8] where social capital is understood as the resources, or mechanisms, (e.g. social support, information channels, social credentials, social control) that inhere within an individual's social and interpersonal networks. These resources can be the property of both the individual and the collective. Szreter and Woolcock [9] summarise this succinctly, where a social cohesion view “…regards social capital as the ‘wires’ (or social infrastructure) while network theorists regard it as the ‘electricity’ (or social resource)” (p. 654). This review takes a social network approach. The contexts of the wires and their electricity flows are also critical to any social capital analysis, especially in terms of how norms, traditions and values mediate the distribution of power [9]. Social capital is often broken down into ‘bonding’ and ‘bridging’ types that cut across both cohesion and network approaches. Bonding social capital refers to strong links within groups with similar characteristics, such as class, race and age. This type of social capital facilitates support between group members and also a level of social influence and control. Bridging social capital describes weaker links between people in groups of more or less equal status and power but with different identities and promotes some types of support, control and collective action [9, 10].

We conducted a systematic review to explore how social capital impacts women’s SRHR in humanitarian settings. We also examined the role of social capital concerning SRHR throughout the disaster management cycle that is currently unexplored in the literature. A clear understanding of the mechanisms and pathways through which social capital plays out in these settings could inform SRH responses in crisis settings. For example, in the context of implementing the MISP, this understanding could help to both ensure it is harnessing existing networks and resources and also anticipating normative resistance and potential deficits in social capital to enable more effective service demand, delivery and access. Incorporating a social capital analysis into SRHR in crisis settings may also strengthen approaches to health system preparedness, including community-based preparedness, and the rebuilding of health systems following crises.

Broader literature agrees that social capital influences health through several mechanisms, the first being the provision of social support [11]. Social support refers to the functional content of relationships [12]. It can be broken down into instrumental support (help to do things), informational support (help to know things), or emotional support (help to feel things) [13]. The second mechanism is social influence through shared norms or informal social control, which refers to the capacity of a community, or smaller group within a community, “to regulate the behaviour of its members according to collectively desired goals” [6, p. 16]. The third mechanism is social engagement, or the enactment of social roles and connections in real-life activities with the aim of promoting the group or network. Social capital can also influence health outcomes by enabling the collective maintenance, or changing, of social norms, facilitating groups to organise and undertake collective action, and by the flow of resources such as information and instrumental support throughout a network [14].

Research into social capital in women’s SRHR in the last decade has also concluded that social capital is an important determinant in health outcomes, and can act through a number of pathways [15–17]. Studies have shown that networks with high levels of education can increase the health related information available to women [18], social support can facilitate access to health services [19], and higher levels of social participation can lead to increased health promoting behaviours [6]. McTavish and Moore [16] found that women in rural Cameroon with stronger social networks and access to resources, such as information or financial capital, within them were more likely to use maternal health care services. They described how, for example, a woman who is a member of a network which values maternal health care is more likely to have access to assistance to access health care services through that network. Story [17] showed that women who lived in Indian communities that had higher numbers of intergroup connections, measured through civic participation in development, religious and cultural groups, were more likely to use antenatal care, professional delivery care and have their children fully immunised. Social capital can also have a negative impact on SRHR. For example, a study in Uganda found that social capital in the form of group norms and values discouraged the use of family planning methods [20]. The research conducted in this area has predominantly used quantitative methods, providing data demonstrating the influence of social capital on SRHR and mapping the associated types, mechanisms and pathways. However, there is a gap in research exploring the intersection of SRH and social capital in humanitarian settings. In this specific area there are few, if any, quantitative studies. As a result, a review of qualitative studies is pertinent as a means of providing a rich description of the known evidence in order to inform future research endeavours.

A consistent critique of the concept of social capital has been the proclivity of researchers to focus on its beneficial impacts without paying sufficient attention to its potential downsides [14]. Portes [21] identified four dimensions of negative social capital including: exclusion of ‘outsiders’ as a result of strong in-group bonding, excess demands placed on some group members to support others, restriction of personal freedoms as a result of informal social control and a downward levelling of norms where close bonding ties hold group members in place making it difficult to mobilise upwards [6, 21]. To understand how social capital can be beneficial and detrimental to health, researchers explored the different impacts of bonding and bridging capital in disadvantaged communities. Studies showed that although bonding capital can be an important survival mechanism in adverse contexts, it can also be a health liability [22, 23]. These studies suggest that it is the presence of bridging capital, the ability of people to access resources beyond their immediate community, which can improve health and wellbeing [6].

In humanitarian contexts, researchers have shown how social capital manifests throughout the different phases of a disaster, from social cohesion enabling community preparedness before a disaster, to immediate support between neighbours during and immediately after a disaster, to how better-connected communities could more effectively mobilise themselves to claim resources during the recovery phase [24, 25]. Aida et al. [24] considered the role of social capital in health following a disaster and reviewed epidemiological studies showing that higher levels of individual social capital were associated with improved mental health outcomes following disasters. These findings are echoed by Noel et al.’s [26] review, which provides evidence for the importance of social capital in post-disaster mental health recovery, though the importance of differentiating between different types and mechanisms of social capital is noted as not all have a positive impact. Despite increasing attention on the role of social capital in post-disaster mental health and humanitarian contexts, few studies investigate physical health [24], including SRH. In particular, there is a dearth of qualitative research exploring social capital and health in general but even more so in relation to SRHR in humanitarian settings [27]. Although there are some qualitative studies that explore elements of social capital, such as social support or social networks (for example, [12–14]), very few explicitly employ a broader social capital framework.

## Methods

This meta-ethnography of qualitative evidence sought to address the review question: what role does social capital play in women’s SRHR in humanitarian settings? The review followed the preferred reporting items for systematic reviews and meta analyses (PRISMA) Statement [28], ENTREQ recommendations for reporting [29] and is registered with PROSPERO, under the registration number CRD42021232396.

### Search strategy and study selection

We conducted an initial scoping exercise to identify appropriate databases and generate a comprehensive set of search terms. Previous systematic reviews involving social capital, SRHR or humanitarian settings were also consulted to expand further and develop the search terms. Six databases were searched, including Medline, Embase, CINAHL, Psycinfo, Proquest and Web of Science. Search strategies are provided as supplementary information to this paper (Additional files 1, 2, 3, 4, 5, 6). Only studies conducted in Low and Lower-Middle-Income Countries (LLMIC) as per the World Bank 2019–2020 classification were included in the review [30].

This review focused on the experiences of women, friends, family, community leaders and health workers, providing both emic and etic perspectives on the subject. Outcomes of interest included anything that was facilitated or hindered by belonging to formal or informal social networks and that influenced women in relation to their SRHR. Studies were included that provided examples of any form of social capital, positive or negative, understood through a network perspective as outlined above.

The review included English-language, peer-reviewed studies published from 1999, when the *Reproductive Health in Refugee Situations: An Inter-Agency Field Manual* was first published (republished in 2010 as the *Inter-Agency Field Manual on Reproductive Health in Humanitarian Settings*) up until the time of searching (March 2021). The manual provided a shared point of reference for the international community and consistently highlights the importance of, and need for, identifying and utilising community networks, knowledge and other social capital related resources to strengthen response programming.

Following the literature search, we combined results and removed duplicates using EndNote. We identified an additional three studies through manually searching bibliographies. After removing duplicates, we used Covidence software to screen titles and abstracts and following this, came to a consensus on studies to be included in a full-text review.

### Data extraction and synthesis

All relevant data in the finding sections of the included papers was extracted for analysis into an excel spreadsheet data extraction form. This included both direct participant quotations and author descriptions that related to the outcomes of interest. We used a meta-ethnography approach, as outlined by Noblit and Hare [31], to analyse the data. This method was chosen as it is frequently used for qualitative analysis in health care research and provides a systematic approach whilst still maintaining the interpretive qualities of the data. This approach is appropriate for this review, concerned with the unique experiences of women in humanitarian settings, as it allows for the non-homogeneity of contextually-based data [32, 33] and enables us to identify examples of social capital in studies that did not necessarily use it as a conceptual framework. As guided by Noblit and Hare’s [31] process, we coded and organised the extracted data from each paper into concepts according to the nature of the social process being described [34]. These concepts were clustered into several groups and given descriptive headings that were used to develop a translations table. Using this table, the findings under each heading for each paper were studied, compared to each other and summarised, producing a synthesis of primary author interpretations. From this, we drew out the main points to form ‘reciprocal translations’ or, third order constructs, agreement on which was reached through discussion with all authors. At this point, a social capital framework and terminology was applied to the findings to interpret and describe the relationships between the third-order constructs and to develop a conceptual model to illustrate those relationships.

### Quality assessment

All included papers were critically appraised by two authors using the Critical Appraisal Skill’s Programme (CASP) assessment tool for qualitative research [35]. All of the studies met most of the criteria outlined on the CASP checklist. However, only five clearly outlined their methodologies, identifying and describing guiding methodological frameworks and methods. The remaining 14 studies identified methods but did not explain their broader methodological approaches. No studies were excluded through this process, consensus on which was reached among all authors.

## Findings

**Fig. 1:**
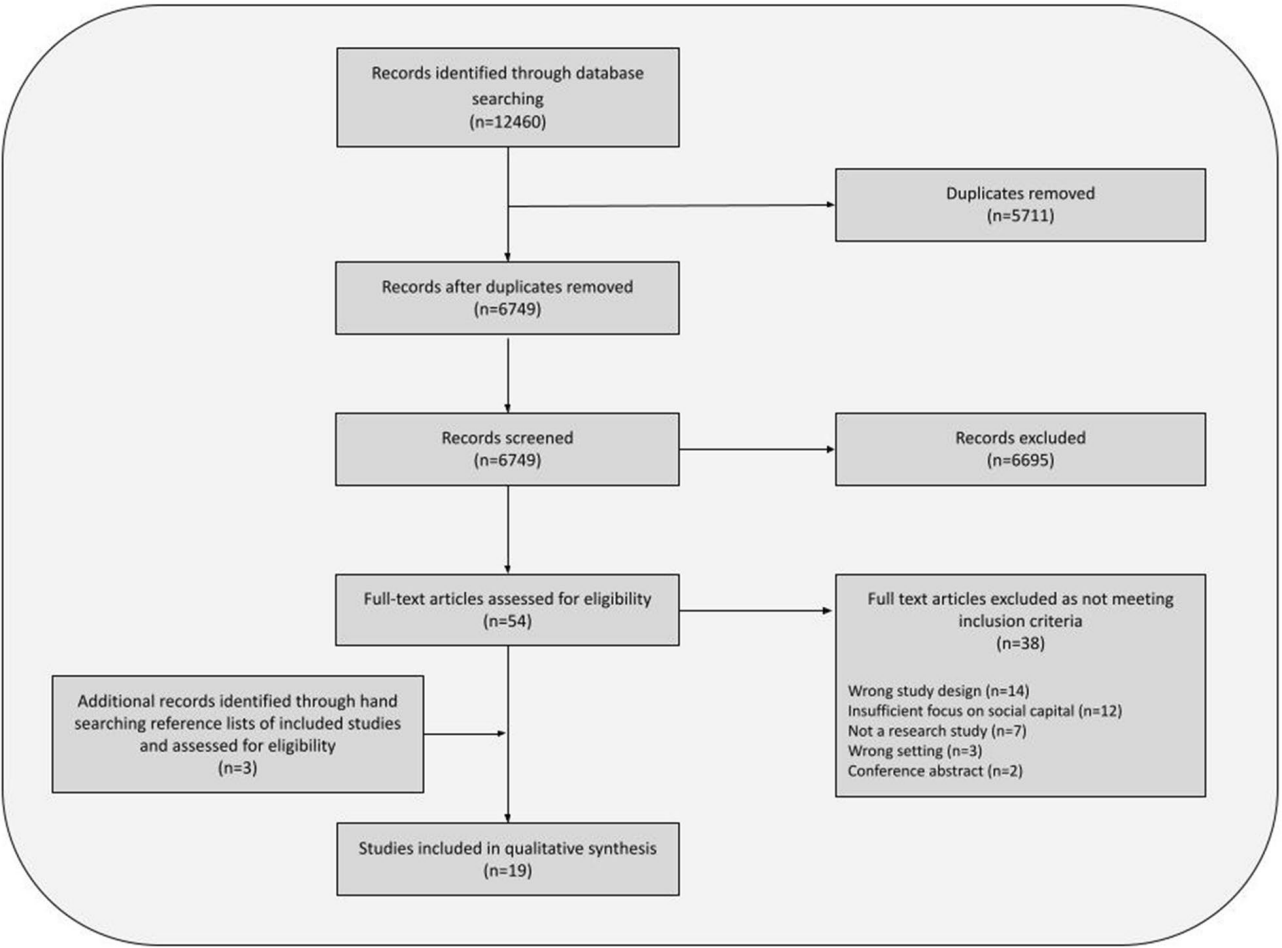
PRISMA flow-chart showing database search and study selection results

Our initial search returned 6749 studies of which 6695 were removed during title and abstract screening. Studies were removed at this stage because they were not research papers, not qualitative or did not consider any aspects of social capital. We conducted a full-text review on 54 papers, from which we identified 16 studies to be included in the analysis. An additional three studies were identified through hand searching the reference lists of included papers, assessed for eligibility and also included for analysis, bringing the total to 19. Figure 1 shows the results of the database search ﻿1 and study selection process.

As outlined in Table 1, fourteen of the included studies were from Africa, four from Asia and one from the Middle East. The SRHR focus of the studies included intimate partner violence, sexual violence, maternal health, family planning, abortion-related care, and HIV. All the studies were conducted in conflict-related humanitarian contexts except for one study undertaken in a flooded region of Cambodia [36].

**Table 1:** Characteristics of studies included in review

References	Country	SRH focus	Humanitarian context	WB FCS*
Badal et al. [37]	Somaliland	Maternal health—ANC	Protracted, ongoing conflict, significant internal displacement Research conducted in two IDP camps	X
Dossa et al. [38]	Democratic Republic of Congo	Sexual violence	Protracted, ongoing conflict, significant internal displacement	X
Elmusharaf et al. [39]	South Sudan	Family planning/abortion related care	Protracted, ongoing conflict, significant internal displacement	X
Guruge et al. [40]	Sri Lanka	IPV	Post-conflict, recovery since end of civil war in 2009. At time of research 70,000 people were still displaced	
Horn [41]	Kakuma Refugee Camp, Kenya	IPV	Kakuma Refugee Camp. At time of research the camp was home to 96,000 refugees from nine countries, the majority from Sudan and Somalia	
Horn et al. [42]	Sierra Leone and Liberia	IPV	Sierra Leone: Post-conflict recovery since end of civil war in 2002 Liberia: Post-conflict recovery since end of civil war in 2003	X
Koegler et al. [43]	Democratic Republic of Congo	Sexual violence	Protracted, ongoing conflict, significant internal displacement	X
Kohli et al. [44]	Democratic Republic of Congo	Sexual violence	Protracted, ongoing conflict, significant internal displacement	X
Kohli et al. [45]	Democratic Republic of Congo	IPV	Protracted, ongoing conflict, significant internal displacement	X
Muzyamba [46]	Democratic Republic of Congo	HIV	Protracted, ongoing conflict, significant internal displacement	X
Rhine [47]	Northern Nigeria	HIV	Ongoing communal and ethno-religious conflict	X
Saulnier et al. [36]	Cambodia	Maternal health	Acute onset flooding response	
Stark et al. [48]	Northern Uganda	Sexual violence	Post-conflict, recovery since the end of conflict in 2006 Research conducted in IDP camps	
Steven et al. [49]	Democratic Republic of Congo	Family planning/abortion related care	Protracted, ongoing conflict, significant internal displacement	X
Strang et al. [50]	Kurdistan, Northern Iraq	IPV	Protracted, ongoing conflict, significant internal displacement Research conducted in an informal IDP camp and neighbouring settlement	X
Teela et al. [51]	Myanmar	Maternal health	Protracted, ongoing conflict	X
Tol et al. [52]	Eastern Uganda	Maternal health—mental health	Post-conflict, recovery since the end of conflict in 2006	
Walstrom et al. [53]	Rwanda	HIV	Post-conflict, recovery since end of war in 1994	
Wild et al. [54]	Timor-Leste	Maternal health—birth choices	Post-conflict, recovery since gaining independence in 2002	X

*WB FCS: The World Bank list of Fragile and Conflict Affected Situations (FCS) is produced annually and provides a classification of low- and middle-income countries that are affected by fragility and conflict. These studies appeared on the list in the year the research was conducted (The World Bank, 2021)

*ANC* antenatal care, *IDP* internally displaced person, *IPV* intimate partner violence

The analysis revealed social capital mechanisms that influenced women’s sexual and reproductive health that were primarily social support, including instrumental, informational and emotional, informal social control and a few examples of collective action; each of these is described below. These mechanisms were largely driven through bonding, and some bridging social capital and led to a number of outcomes that had the potential to both facilitate and hinder women’s SRHR. The nature of the outcomes was mediated by the values, norms and traditions held by network members. It should be noted that other social capital mechanisms may play a role in women’s SRHR in humanitarian settings. However, these findings only represent those mechanisms that were identified in the included studies.

### Instrumental social support

One of the forms of instrumental support provided by community members was in playing a counselling and mediating role for women experiencing intimate partner violence (IPV) [41, 42, 45, 50]. A hierarchy of response was consistently described across the studies where women experiencing IPV would first go to family members for advice and counsel and then, if the issue was not resolved, to community leaders. A Yezidi woman outlined this process, “First I go to my daughter and try and resolve it with my husband. Then we go to the head of the household. If that doesn’t work, we go to Baba Sheikh…" [50, p. 9]*.* Beyond this, in some cases, the issue would be taken outside of the community.

"Elders will solve the problem… If he doesn’t stop, the elders can take the case to the chairman, and if he can’t solve it he will call security, who take the woman to Gender and Social Services" ([41, p. 164] Somali Refugee)

Whilst these community processes for responding to IPV could be effective and protective mechanisms for women, they were often guided by male-dominated social norms and cultural traditions which prioritised keeping the family together over the safety and wellbeing of the woman involved. This is demonstrated in the following quote where a typical response, by parents or in-laws, to a woman seeking help for IPV was described.

“They usually judge the case, and if you the woman are right, they usually beg you and ask you to please remain in that home. They get angry with the man and warn him strongly not to repeat. Then you go back home and continue.” [42, p. 113]

Horn et al. [42] also noted that where violence continued, women sometimes returned to their parents’ household temporarily or permanently, although this was not always a possibility. The findings from these studies show how this social capital mechanism relating to community and family responses to IPV has the potential to impact women’s health both positively and negatively, depending on the shared norms prevalent in the bonded networks it emerges from.

Another form of instrumental support identified in several studies was linking and referring women to formal institutions outside the immediate community. The studies reviewed included community members and leaders referring women to health services concerning induced abortion care, sexual violence, family planning and antenatal care [36, 48, 49, 52].

The sharing of food and house or farm work was a common form of instrumental support described in studies investigating formal group networks such as HIV and sexual violence support groups. These groups often filled a gap for people who were isolated from their families and communities, providing essential day-to-day support.

“…When we are here we make a special kind of friendship. Suppose one of us fell sick, we go see her, we prepare food for her, try to console her and discuss what we can do to make her better. And when we go to see her in her area, no one will ask why we come, we go there to visit as a friend, not as an HIV-positive person.” [53, p. 10]

One of the HIV support groups filled a matchmaking gap, helping members navigate the social requirements for finding a spouse that would usually be conducted by family members.

"I enrolled [in this group] just to get a husband to marry...The chairman…found me a husband at [the hospital]…We met and although he was set to be introduced to some other girl, on seeing me he liked me” [47, p. 13]

### Informational social support

Informational social support crosses over somewhat with the linking and referring aspect of instrumental social support. The studies reviewed showed that women had access to sources of information regarding SRHR issues in their communities. This included information regarding illness during pregnancy [52], place of birth and strategies for labour [36, 54], perinatal care [36, 37], mental health, HIV [46, 53], abortion-related care and family planning [49]. For example, Steven et al. [49] documented how community leaders advised women who had terminated their pregnancy of the need to visit a health facility for post-abortion care, or village chiefs who promoted the value of prenatal care in a Cambodian study by Saulnier et al. [36].

However, the provision of information was not always consistent with promoting health outcomes for women. For example, some studies described how respected elders provided information regarding antenatal care suggesting it would cause complications in pregnancy and lead to needing a caesarean section [37]. A study in Timor Leste showed how women drew on knowledge and tradition handed down through generations of mothers and grandmothers regarding place of birth: "Because it’s like what happened a long time ago, the grandmothers had their baby at home so we will do it just like the grandmothers" [54, p. 2041].

One of the reasons women gave for choosing to birth at home without skilled care, rather than at a health facility, was that traditional knowledge promoted birth occurring in an upright position, physically supported by family members. Health facilities did not allow more than one person to attend a woman’s birth and this influenced women’s and families’ decisions to birth at home.

"I think giving birth at home is better than at the health centre because sometimes the midwife doesn’t allow the husband or the parents to go in the room and there is nobody we can hold onto. In the house we have the husband and the parents to hold onto." [54, p. 2043]

This example highlights the complexity of how social capital impacts women’s SRHR. Here, the knowledge resource accessed by pregnant women encouraged the higher risk option of delivering their babies at home, without skilled care.

### Emotional social support

Emotional support came primarily from close family or friend networks, and especially from mothers. This was particularly prominent in studies investigating IPV and other forms of gender-based violence (GBV) [43, 48, 50], where affected women who were subject to stigma and shame from their broader communities found critical solace and safety in close relationships. A displaced Yezidi woman experiencing IPV described the nature of this social support, “When I cannot stand my miserable life then I go to speak to my mother to feel comfortable. I feel safe and free when I speak with her” [15, p. 9].

Another study in Uganda showed how women drew on emotional support from family and friends when experiencing poor maternal mental health [52] and a study conducted in Timor Leste emphasised the importance of emotional support from family and friends during labour and childbirth [54].

Studies that investigated formal group settings such as support groups for people living with HIV or affected by sexual violence found a high level of emotional support was provided through these [38, 53]. Participants referred to their fellow group members as family and friends, emphasising the significant impact of this form of emotional support on their mental and physical health, such as in the following quotes: "When I’m with the other women who have also been raped, I also feel stronger." [38, p. 251].

“…Before I came I was suffering from headaches—every day I was suffering from headaches—and then after meeting with counsellors and the other women, we’ve shared our experience and now I’m feeling better”. [53, p. 7]

Group participants often faced isolation from their families and friends due to stigma around issues such as HIV or being a rape survivor. This meant they could risk losing the social support they would have had from those sources, increasing the importance of this source of social capital. The support groups researched in the studies relied on bonding ties which enabled members to connect around shared experiences. The groups also enabled bridging ties, which may have been previously lost, back into their communities [53]. One woman recounted how the emotional support and safety accessed through participation in a support group enabled an improved sense of social functioning which led to reconnection and interaction with her broader community:

Nowadays after coming to the group I don’t have any conflict with neighbours. I know how to handle them. I’m feeling comfortable when I talk to them and it’s because of the support group” [53, p. 7]

### Informal social control

Informal social control was a common theme throughout many of the included studies in the areas of gender-based violence, abortion-related care, family planning and maternal health [36, 37, 39–41, 45, 50]. Informal social control has typically been seen as a mechanism that bought about positive impacts to community members at large however the majority of the examples identified in the studies had potentially adverse impacts on women’s health or rights [39–41, 45, 49, 50]. By way of example, Elmusharaf et al. [39] showed how deeply entrenched social norms created pressure and expectation to have large families. Women who did not, or were not able to, have large numbers of children were subject to stigma and shame, as seen in the following quote: "If a lady doesn’t bring a child that means she’s not good and people don’t like her." [39, p. 5].

Stigma and shame were central in driving the process of informal social control in other contexts also, for example where women faced significant social pressure to keep unwanted pregnancies.

"A woman who terminates her pregnancy … she is considered a criminal, she is discriminated against, she is considered as the one who does not have friends because she is a murderer" [49, p. 5]

Informal social control also played a role in the way communities responded to IPV, which as previously mentioned, did not always prioritise the safety and wellbeing of the women involved. The risk of possible humiliation and entailing consequences created social pressure to remain in violent relationships [40, 41, 45, 50].

"The violence is happening but I tell myself it is not worth it and I try to keep silent. He beats me but we are IDPs, it would be better to keep silent than making the problem bigger than its normal size." [50, p. 7]

Women survivors of sexual violence in humanitarian settings also experienced discrimination and this sometimes influenced their decisions to disclose the violence and seek the support they needed [38, 48].

“It’s a secret for us. If you approach us, we’ll talk to you and you’ll discover the problem we’re living with. But if you don’t come to us, it’s not easy for us to stand up, like that, in front of a lot of people, or the assembly, and start saying, “I was raped.” It’s not easy.” [38, p. 250]

Social norms around the ownership of women by their husbands and husbands’ families were common throughout the studies. They helped facilitate informal social control around some SRH issues. The findings demonstrated that this could result in both positive and negative impacts on health behaviours. For example, Badal et al. [37] described how husband and family members could influence women to either attend or not attend antenatal care depending on their beliefs. Saulnier et al. [36] similarly showed that women were expected to do as their husband and family chose in relation to where to deliver their babies, whether this be with or without skilled care. Regardless of the impact, however, in each of these examples women had minimal agency in their SRH-related decisions.

### Collective action

Though not as common in the reviewed studies as the two other social capital mechanisms, there were also two examples of collective action. Both examples were in the context of support groups, which used their collective voice to advocate, seeking to change norms and attitudes that had caused them harm [42, 46]. An HIV-positive IDP from Congo describes this in the following quote.

“We now work in unit to challenge the bad treatment, stigma and discrimination which we have been subjected to for a long time. We now speak as one voice.” [46, p. 3]

Muzyamba [46] explained how the support groups could challenge issues such as patriarchy and sexual cleansing. Horn et al. [42] described the power of collective action in the context of IPV. As a group, women could confront a man concerning his violent behaviour that was often effective in bringing about change.

## Discussion

**Fig. 2:**
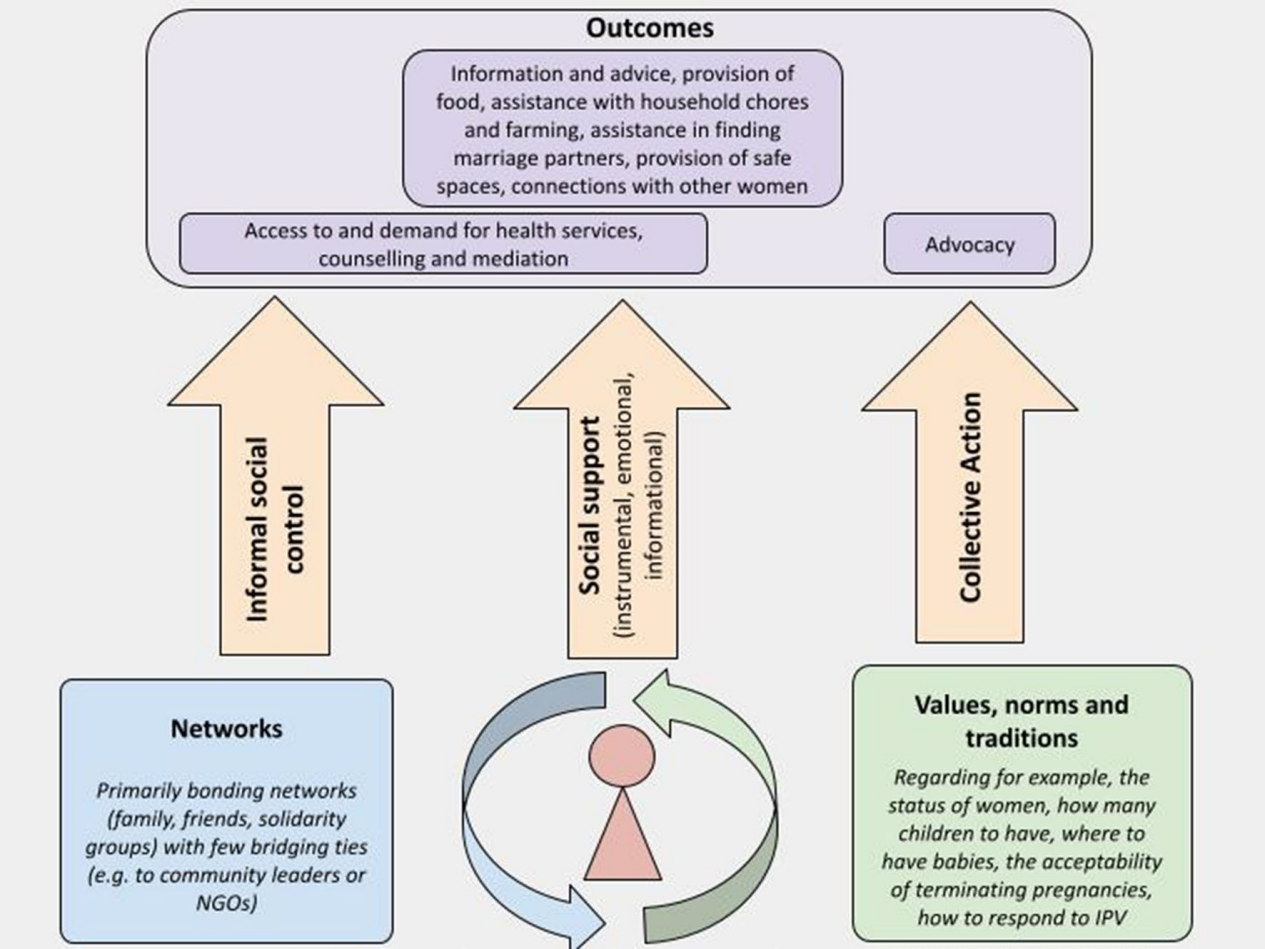
Relationships between social capital and outcomes identified in reviewed studies

This review found that women in the included studies belonged to interconnected family and community networks and in some cases, formal group networks. These networks were primarily connected with bonding ties, and some bridging ties to broader community networks. The ties were reinforced by a set of values, norms and traditions which guide how women and communities respond to women’s SRHR. In the included studies, the three mechanisms through which social capital played a role in women’s SRHR were social support, informal social control, and collective action, resulting in several outcomes. Figure 2 provides an overview of the types of networks, social capital pathways and outcomes identified in this review. These outcomes included access to and demand for health services, information and advice, provision of food, assistance with household chores and farming, safe spaces, connections with other women, counselling, mediation, assistance in finding marriage partners and advocacy. The outcomes were mediated by the prevailing values, norms and traditions, including the status of women and their decision-making power. As a result, the outcomes had the potential to have both beneficial, and in some cases, adverse impacts on women’s SRHR. It should be noted that all the participants across the studies were of a similar socio-economic status so whilst this is a significant consideration in social capital analysis, its impact on outcomes was not variable.

The review findings are consistent with existing research that shows that social capital can play an important role in influencing health in humanitarian settings [24–26]. They also echo previous research, which has explored the specific pathways through which social capital influences health throughout the different phases of the disaster management cycle [24, 25, 55]. Most of the reviewed studies were conducted in the relief and recovery phases. They showed how social capital enables a bottom-up approach where community members provide instrumental, informational and emotional support for one another. The few examples of bridging capital in the findings were also consistent with broader research demonstrating its necessity in opening up “pathways to longer-term survival and wider…community recovery” [24, p. 174]. Previous research has distinguished between existing social capital and new social capital, suggesting that literature to date has focussed mainly on the value of existing social capital in relation to disaster mitigation and recovery and the creation of new social capital concerning preparedness and response [25]. In line with this, the review findings affirmed the importance of existing social capital in the context of the recovery phase. In contrast however, our review also highlighted the value of new social capital built through support groups during the recovery phase.

In the context of the existing literature, the review findings demonstrate the value in considering the role of social capital in women’s SRHR across all phases of the disaster management cycle including an exploration of how the specific mechanisms and types may impact SRH outcomes. Applying a social capital framework to planning and coordination of SRH programming, such as the MISP for SRH, could provide valuable guidance in how to harness the potential of existing networks and resources. This application could also help to identify where critical gaps exist and develop appropriate interventions to address them. Importantly this should include an individual level of social capital analysis that considers the differential access to social capital *within* networks, and how the social capital of some network members can limit the potential of others, as seen in the review findings. Not including this level of analysis risks implementing health interventions that are not as effective as possible or, at worst, that reproduce forms of social capital that further constrain women's agency. This analysis should identify whose voices need to be ‘in the room’ when it comes to community consultation, and program implementation, developing the bridging social capital required for communities, and especially women, to build sustainable pathways to improved SRH.

In the context of the global COVID-19 pandemic the potential value of incorporating social capital into the analysis and response to crises is even more salient. Projections that warn of the serious impact that the pandemic could have on access to life-saving SRH services are now beginning to be borne out [56, 57]. Emerging research points towards the importance of considering social capital in responding to and living with COVID-19, especially in ensuring that the most vulnerable and marginalised members of society are informed about existing services and have access to the services they need when they need them [58]. Social capital could play a critical role in helping to minimise the immediate impact of service demand and disruptions, social isolation, and a weakened health system on SRHR. In the longer term, it could help facilitate the recovery of local health systems that deliver equitable access to SRH services and that are built on robust connections with the communities and people they are designed to serve.

### Limitations and future research

A significant limitation of this review was the lack of studies that explicitly applied a social capital approach. Only one of the 19 studies reviewed used social capital as an explanatory framework [50]. When selecting studies to be included in the review the authors searched the studies for examples of social capital interventions, using a common definition of social capital, as outlined in the background of this paper. We addressed the potential for subjectivity through this process as far as possible by ensuring agreement from all authors regarding what constituted social capital in each paper. As most of the included studies did not set out to specifically identify or measure the role of social capital in women’s SRHR in humanitarian settings, it is likely that there are some gaps in the picture this review presents. Another limitation lies in the heavy weighting of included studies towards conflict-related humanitarian settings, with only one conducted in a natural disaster context. Though both humanitarian, there are differences in some of the issues, for example, conflict-driven sexual violence, which may be more prevalent in one than the other. The small sample of studies in natural disaster settings did not enable any themes or patterns to be identified specific to that context. There is a need for further research that explores the link between social capital and women’s SRHR in humanitarian contexts, especially natural disaster settings. This review was also limited to peer-reviewed studies, and future research could benefit from exploring grey literature.

This review highlights a significant area for further research, exploring how a social capital analysis can be incorporated into SRHR crisis preparedness, response, mitigation and recovery efforts. As part of addressing this question, research is needed to identify the pathways through which social capital influences SRHR in humanitarian settings. Whilst this review elicited some insights, further research is required to provide a more complete picture which would reveal the barriers and opportunities, relating to social capital in strengthening the demand for and quality of SRH services in humanitarian settings. Another important element of future research, which was not explicitly addressed in this review, would be investigating how crises break down social capital in relation to SRHR and how the health of women, who have lost most, or all of their social capital, is impacted.

## Conclusions

Understanding how social capital influences health in humanitarian settings can offer important insights for professionals and communities in terms of service delivery, access and demand, from preparedness planning through to response and recovery. To date, little research has been conducted that focuses specifically on SRHR and future studies would be of great benefit to enhancing SRH responses in crisis settings. A more comprehensive picture of the relationships between social capital and SRHR in humanitarian contexts could enable more effective design and implementation of interventions resulting in improved SRHR for women in crisis settings worldwide.

## Supplementary Information

**Additional file 1.** CINAHL Search Strategy.

**Additional file 2.** Embase Search Strategy.

**Additional file 3.** Medline Search Strategy.

**Additional file 4.** Proquest Search Strategy.

**Additional file 5.** PsychInfo Search Strategy.

**Additional file 6.** Web of Science Search Strategy.

## Data Availability

All data is in the public domain. Samples of search strategies are available in the supplementary material.

## Abbreviations

MISP: Minimum initial service package
SRH: Sexual and reproductive health
SRHR: Sexual and reproductive health and rights
PRISMA: Preferred reporting items for systematic reviews and meta-analyses
LLMIC: Low and lower-middle income countries
CASP: Critical appraisal skills programme
WB FCS: The World Bank list of Fragile and Conflict Affected Situations
ANC: Antenatal care
IDP: Internally displaced person
IPV: Intimate partner violence
GBV: Gender-based violence
