# Supplementary material for: The role of social capital in women’s sexual and reproductive health and rights in humanitarian settings: a systematic review of qualitative studies
Source: Confl Health. 2021 Nov 24;15:87. doi: 10.1186/s13031-021-00421-1 (PMC8611620; doi:10.1186/s13031-021-00421-1)
Supplement: Supplementary file 2 — Additional file 2. Embase Search Strategy. [file 13031_2021_421_MOESM2_ESM.docx]

Embase (2186)

( “Sexual Health”) OR (MH “Reproductive Health) OR (MH “Pregnancy”) OR (“Pregnant Woman”) OR (Adolescent Pregnancy”) OR (“Pregnancy Termination”) OR (“Unwanted Pregnancy”) OR (“Unplanned Pregnancy”) (MH “Family Planning+”) OR (MH “Contraception+”) OR (MH “Abortion, Induced+”) OR (MH “Prenatal Care”) OR (MH “Postnatal Care+”) OR (MH “Perinatal Care”) OR (MH “Human Immunodeficiency Virus+”) OR (MH “Acquired Immunodeficiency Syndrome”) OR (MH “Sexually Transmitted Diseases+”) OR (“Maternal-Child Health”) OR (MH “Maternal Health Services+”) OR (MH “Gender-Based Violence) OR (MH “Intimate Partner Violence+”) OR (MH “Sexual Abuse+”) OR (MH “Reproductive Rights”) OR (MH “Women’s Health”) OR (“sexual and reproductive health”) OR (“sexual health”) OR (“reproductive health”) OR (pregnan*) OR (“family planning”) OR (contracept*) OR (abortion) OR (“prenatal healthcare”) OR (“prenatal care”) OR (“postnatal care”) OR (“postnatal healthcare”) OR (“(perinatal care”) OR (“perinatal healthcare”) OR (“antenatal care”) OR (“antenatal healthcare”) OR (HIV) OR (“HIV/AIDS”) OR (AIDS) OR (STIs) OR (“maternal and newborn health”) OR (“maternal health”) OR (“gender-based violence”) OR (“spouse abuse”) OR (“intimate partner violence”) OR “reproductive rights” OR (“women’s health)

AND

(MH “Social Capital”) OR (MH “Social Norms”) OR (MH “Social Networks”) OR (MH “Social Networking+”) OR (MH Community Networks”) OR (“social capital”) OR (“social cohesion”) OR (“social norm*) OR (“social network*”) OR (“social support*”) OR (“collective N3 efficacy”)

AND

(MH “Humanitarian Aid+”) OR (MH “Disasters+”) OR (MH “Natural Disasters+”) OR (MH “Mass Casualty Incidents”) OR (MH “War”) OR (MH “Refugees+”) OR (MH “Refugee Camps”) OR (MH “Disease Outbreaks+”) OR (MH “Zika Virus”) OR (MH “Ebola Virus”) OR (MH “SARS Virus”) OR (MH “Severe Acute Respiratory Syndrome”) OR (MH “Middle East Respiratory Syndrome Coronavirus”) OR (MH “Middle East Respiratory Syndrome) OR (MH “COVID-19”) OR (humanitarian) OR (emergency) OR (emergencies) OR (disaster*) OR (crisis) OR (crises) OR (avalanche*) OR (cyclone*) OR (drought*) OR (earthquake*) OR (flood*) OR (hurricane*) OR (landslide*) OR (“tidal wave*”) OR (tsunami*) OR (typhoon*) OR (conflict*) OR (war*) OR (“fragile state*”) OR (warfare) OR (refugee*) OR (“disease outbreak*”) OR (epidemic*) OR (pandemic*) OR (zika) OR (ebola) OR (SARS) OR (“Severe Acute Respiratory Syndrome”) OR (MERS) OR (“Middle East Respiratory Syndrome”) OR ("COVID-19") OR ("COVID 19") OR ("COVID 2019") OR ("severe acute respiratory syndrome coronavirus 2") OR ("SARS-CoV-2") OR ("2019-nCoV") OR ("2019 ncov") OR ((novel OR new OR "2019" OR wuhan OR hubei OR china) AND (coronavirus OR covid))
