# Supplementary material for: The role of social capital in women’s sexual and reproductive health and rights in humanitarian settings: a systematic review of qualitative studies
Source: Confl Health. 2021 Nov 24;15:87. doi: 10.1186/s13031-021-00421-1 (PMC8611620; doi:10.1186/s13031-021-00421-1)
Supplement: Supplementary file 3 — Additional file 3. Medline Search Strategy. [file 13031_2021_421_MOESM3_ESM.doc]

Medline (1709)

1 exp Sexual Health/

2 exp Reproductive Health/

3 exp Reproductive Health Services/

4 exp Pregnancy/

5 exp Pregnant Women/

6 exp Family Planning Services/

7 exp Contraception/

8 exp Abortion, Induced/

9 exp Prenatal Care/

10 exp Postnatal Care/

11 exp Perinatal Care/

12 exp Midwifery/

13 exp Acquired Immunodeficiency Syndrome/

14 exp HIV/

15 exp Sexually Transmitted Diseases/

16 exp Maternal Health Services/

17 exp Maternal Health/

18 exp Gender-Based Violence/

19 exp Spouse Abuse/

20 exp Intimate Partner Violence/

21 exp Reproductive Rights/

22 exp Women's Health/

23 "sexual and reproductive health".tw.

24 "sexual health".tw.

25 "reproductive health".tw.

26 pregnan*.tw.

27 "family planning".tw.

28 contracept*.tw.

29 abortion.tw.

30 ("prenatal healthcare" or "prenatal care").tw.

31 ("postnatal healthcare" or "postnatal care").tw.

32 ("perinatal healthcare" or "perinatal care").tw.

33 ("antenatal healthcare" or "antenatal care").tw.

34 AIDS.tw.

35 HIV.tw.

36 "HIV/AIDS".tw.

37 STIs.tw.

38 "maternal and newborn health".tw.

39 "maternal health".tw.

40 "gender-based violence".tw.

41 "spouse abuse".tw.

42 "intimate partner violence".tw.

43 "reproductive rights".tw.

44 "women's health".tw.

45 1 or 2 or 3 or 4 or 5 or 6 or 7 or 8 or 9 or 10 or 11 or 12 or 13 or 14 or 15 or 16 or 17 or 18 or 19 or 20 or 21 or 22 or 23 or 24 or 25 or 26 or 27 or 28 or 29 or 30 or 31 or 32 or 33 or 34 or 35 or 36 or 37 or 38 or 39 or 40 or 41 or 42 or 43 or 44 (1627752)

46 exp Social Capital/

47 exp Social Norms/

48 exp Social Networking/

49 exp Community Networks/

50 exp Social Support/

51 exp Self-Help Groups/

52 "social capital".tw.

53 "social cohesion".tw.

54 "social norm*".tw.

55 "social network*".tw.

56 "social support*".tw.

57 (collective adj3 efficacy).tw.

58 46 or 47 or 48 or 49 or 50 or 51 or 52 or 53 or 54 or 55 or 56 or 57 (127992)

59 exp Relief Work/

60 exp Emergency Shelter/

61 exp Disasters/

62 exp Natural Disasters/

63 exp Disaster Victims/

64 exp Avalanches/

65 exp Cyclonic Storms/

66 exp Droughts/

67 exp Earthquakes/

68 exp Floods/

69 exp Landslides/

70 exp Tidal Waves/

71 exp Tsunamis/

72 exp "Warfare and Armed Conflicts"/

73 exp Warfare/

74 exp Refugees/

75 exp Refugee Camps/

76 exp Disease Outbreaks/

77 exp Epidemics/

78 exp Pandemics/

79 exp Zika Virus/

80 exp Zika Virus Infection/

81 exp Ebolavirus/

82 exp SARS Virus/

83 exp Middle East Respiratory Syndrome Coronavirus/

84 humanitarian.tw.

85 (emergency or emergencies).tw.

86 disaster*.tw.

87 (crisis or crises).tw.

88 (avalanche* or cyclone* or drought* or earthquake* or flood* or hurricane* or landslide* or "tidal wave*" or tsunami* or typhoon*).tw.

89 conflict*.tw.

90 war*.tw.

91 "fragile state*".tw.

92 warfare.tw.

93 refugee*.tw.

94 "disease outbreak*".tw.

95 epidemic*.tw.

96 pandemic*.tw.

97 zika.tw.

98 ebola.tw.

99 SARS.tw.

100 "Severe Acute Respiratory Syndrome".tw.

101 MERS.tw.

102 "Middle East Respiratory Syndrome".tw.

103 ("COVID-19" or "COVID 19" or "COVID 2019" or "severe acute respiratory syndrome coronavirus 2" or "SARS-CoV-2" or "2019-nCoV" or "2019 ncov" or ((novel or new or "2019" or wuhan or hubei or china) and (coronavirus or covid))).tw.

104 59 or 60 or 61 or 62 or 63 or 64 or 65 or 66 or 67 or 68 or 69 or 70 or 71 or 72 or 73 or 74 or 75 or 76 or 77 or 78 or 79 or 80 or 81 or 82 or 83 or 84 or 85 or 86 or 87 or 88 or 89 or 90 or 91 or 92 or 93 or 94 or 95 or 96 or 97 or 98 or 99 or 100 or 101 or 102 or 103

105 45 and 58 and 104

106 limit 105 to yr="1999 -Current"

107 limit 106 to (clinical trial, all or meta analysis or randomized controlled trial or "systematic review") (115)

108 106 not 107

***************************
