# Supplementary material for: The role of social capital in women’s sexual and reproductive health and rights in humanitarian settings: a systematic review of qualitative studies
Source: Confl Health. 2021 Nov 24;15:87. doi: 10.1186/s13031-021-00421-1 (PMC8611620; doi:10.1186/s13031-021-00421-1)
Supplement: Supplementary file 4 — Additional file 4. Proquest Search Strategy. [file 13031_2021_421_MOESM4_ESM.docx]

ProQuest (3505)

((noft(humanitarian OR emergency OR emergencies OR disaster* OR crisis OR crises) OR noft(avalanche* OR cyclone* OR drought* OR earthquake* OR flood* OR hurricane* OR landslide* OR ("tidal wave" OR "tidal waves") OR tsunami* OR typhoon*) OR noft(("disease outbreak" OR "disease outbreaks") OR epidemic* OR pandemic* OR zika OR ebola OR SARS OR "severe acute respiratory syndrome" OR MERS OR "Middle East Respiratory Syndrome") OR noft(("COVID-19" OR "COVID 19" OR "COVID 2019" OR "severe acute respiratory syndrome coronavirus 2" OR "SARS-CoV-2" OR "2019-nCoV" OR "2019 ncov" OR ((novel OR new OR "2019" OR wuhan OR hubei OR china) AND (coronavirus OR covid)))) OR noft(conflict* OR war* OR ("fragile state" OR "fragile states") OR warfare OR refugee*)) AND noft("social capital" OR "social cohesion" OR ("social norm" OR "social norms") OR ("social network" OR "social networking" OR "social networks") OR ("social support" OR "social supports")) AND (noft("sexual and reproductive health" OR "sexual health" OR "reproductive health") OR noft(pregnan*) OR noft("family planning" OR contracept* OR abortion) OR noft("prenatal healthcare" OR "prenatal care" OR "postnatal healthcare" OR "postnatal care" OR "perinatal healthcare" OR "perinatal care" OR "antenatal healthcare" OR "antenatal care") OR noft(AIDS OR HIV OR "HIV/AIDS" OR STIs) OR noft("maternal and newborn health" OR "maternal health" OR "women's health") OR noft("gender-based violence" OR "spouse abuse" OR "intimate partner violence") OR noft("reproductive rights")) AND (stype.exact("Scholarly Journals") AND pd(19990101-20210304) AND PEER(yes))) NOT bdl(1007529 10000003 1007428 1007551 1007427 1007426 10000030 1007516 1007603 1007430)
