# Supplementary material for: The role of social capital in women’s sexual and reproductive health and rights in humanitarian settings: a systematic review of qualitative studies
Source: Confl Health. 2021 Nov 24;15:87. doi: 10.1186/s13031-021-00421-1 (PMC8611620; doi:10.1186/s13031-021-00421-1)
Supplement: Supplementary file 5 — Additional file 5. PsychInfo Search Strategy. [file 13031_2021_421_MOESM5_ESM.pdf]

MY
EBSCOhost

Thursday, March 04, 2021 11:09:23 PM

#	Query	Limiters/Expanders	Last Run Via	Results
S73	S70 AND S71 AND S72	Limiters - Publication Year: 1999-2021; Peer Reviewed<br>Expanders - Apply related words; Apply equivalent subjects<br>Search modes - Boolean/Phrase	Interface - EBSCOhost Research Databases<br>Search Screen - Advanced Search<br>Database - APA PsycInfo	1,834
S72	S43 OR S44 OR S45 OR S46 OR S47 OR S48 OR S49 OR S50 OR S51 OR S52 OR S53 OR S54 OR S55 OR S56 OR S57 OR S58 OR S59 OR S60 OR S61 OR S62 OR S63 OR S64 OR S65 OR S66 OR S67 OR S68 OR S69	Expanders - Apply related words; Apply equivalent subjects<br>Search modes - Boolean/Phrase	Interface - EBSCOhost Research Databases<br>Search Screen - Advanced Search<br>Database - APA PsycInfo	460,652
S71	S32 OR S33 OR S34 OR S35 OR S36 OR S37 OR S38 OR S39 OR S40 OR S41 OR S42	Expanders - Apply related words; Apply equivalent subjects<br>Search modes - Boolean/Phrase	Interface - EBSCOhost Research Databases<br>Search Screen - Advanced Search<br>Database - APA PsycInfo	147,489
S70	S1 OR S2 OR S3 OR S4 OR S5 OR S6 OR S7 OR S8 OR S9 OR S10 OR S11 OR S12 OR S13 OR S14 OR S15 OR S16 OR S17 OR S18 OR S19 OR S20 OR S21 OR S22 OR S23 OR S24 OR S25 OR S26 OR S27 OR S28 OR S29 OR S30 OR S31	Expanders - Apply related words; Apply equivalent subjects<br>Search modes - Boolean/Phrase	Interface - EBSCOhost Research Databases<br>Search Screen - Advanced Search<br>Database - APA PsycInfo	218,548
S69	("COVID-19" or "COVID 19" or "COVID 2019" or "severe acute respiratory syndrome coronavirus 2" or "SARS-CoV-2" or	Expanders - Apply related words; Apply equivalent subjects<br>Search modes - Boolean/Phrase	Interface - EBSCOhost Research Databases<br>Search Screen - Advanced Search<br>Database - APA PsycInfo	Display

	"2019-nCoV" or "2019 ncov" or ((novel or new or "2019" or wuhan or hubei or china) and (coronavirus or covid)))			
S68	(MERS OR "Middle East Respiratory Syndrome")	Expanders - Apply related words; Apply equivalent subjects<br>Search modes - Boolean/Phrase	Interface - EBSCOhost Research Databases<br>Search Screen - Advanced Search<br>Database - APA PsycInfo	Display
S67	(SARS OR "Severe Acute Respiratory Syndrome")	Expanders - Apply related words; Apply equivalent subjects<br>Search modes - Boolean/Phrase	Interface - EBSCOhost Research Databases<br>Search Screen - Advanced Search<br>Database - APA PsycInfo	Display
S66	ebola	Expanders - Apply related words; Apply equivalent subjects<br>Search modes - Boolean/Phrase	Interface - EBSCOhost Research Databases<br>Search Screen - Advanced Search<br>Database - APA PsycInfo	Display
S65	zika	Expanders - Apply related words; Apply equivalent subjects<br>Search modes - Boolean/Phrase	Interface - EBSCOhost Research Databases<br>Search Screen - Advanced Search<br>Database - APA PsycInfo	Display
S64	pandemic*	Expanders - Apply related words; Apply equivalent subjects<br>Search modes - Boolean/Phrase	Interface - EBSCOhost Research Databases<br>Search Screen - Advanced Search<br>Database - APA PsycInfo	Display
S63	epidemic*	Expanders - Apply related words; Apply equivalent subjects<br>Search modes - Boolean/Phrase	Interface - EBSCOhost Research Databases<br>Search Screen - Advanced Search<br>Database - APA PsycInfo	Display
S62	"disease outbreak*"	Expanders - Apply related words; Apply equivalent subjects<br>Search modes - Boolean/Phrase	Interface - EBSCOhost Research Databases<br>Search Screen - Advanced Search<br>Database - APA PsycInfo	Display
S61	refugee*	Expanders - Apply related words; Apply equivalent	Interface - EBSCOhost Research Databases	Display

		subjects<br>Search modes - Boolean/Phrase	Search Screen - Advanced Search<br>Database - APA PsycInfo	
S60	warfare	Expanders - Apply related words; Apply equivalent subjects<br>Search modes - Boolean/Phrase	Interface - EBSCOhost Research Databases<br>Search Screen - Advanced Search<br>Database - APA PsycInfo	Display
S59	"fragile state*"	Expanders - Apply related words; Apply equivalent subjects<br>Search modes - Boolean/Phrase	Interface - EBSCOhost Research Databases<br>Search Screen - Advanced Search<br>Database - APA PsycInfo	Display
S58	war*	Expanders - Apply related words; Apply equivalent subjects<br>Search modes - Boolean/Phrase	Interface - EBSCOhost Research Databases<br>Search Screen - Advanced Search<br>Database - APA PsycInfo	Display
S57	conflict*	Expanders - Apply related words; Apply equivalent subjects<br>Search modes - Boolean/Phrase	Interface - EBSCOhost Research Databases<br>Search Screen - Advanced Search<br>Database - APA PsycInfo	Display
S56	(avalanche* OR cyclone* OR drought* OR earthquake* OR flood* OR hurricane* OR landslide* OR "tidal wave*" OR tsunami* OR typhoon*)	Expanders - Apply related words; Apply equivalent subjects<br>Search modes - Boolean/Phrase	Interface - EBSCOhost Research Databases<br>Search Screen - Advanced Search<br>Database - APA PsycInfo	Display
S55	crisis OR crises	Expanders - Apply related words; Apply equivalent subjects<br>Search modes - Boolean/Phrase	Interface - EBSCOhost Research Databases<br>Search Screen - Advanced Search<br>Database - APA PsycInfo	Display
S54	disaster*	Expanders - Apply related words; Apply equivalent subjects<br>Search modes - Boolean/Phrase	Interface - EBSCOhost Research Databases<br>Search Screen - Advanced Search<br>Database - APA PsycInfo	Display
S53	emergency OR emergencies	Expanders - Apply related words; Apply equivalent subjects	Interface - EBSCOhost Research Databases<br>Search Screen - Advanced	Display

		Search modes - Boolean/Phrase	Search<br>Database - APA PsycInfo	
S52	humanitarian	Expanders - Apply related words; Apply equivalent subjects<br>Search modes - Boolean/Phrase	Interface - EBSCOhost Research Databases<br>Search Screen - Advanced Search<br>Database - APA PsycInfo	Display
S51	DE "Coronavirus"	Expanders - Apply related words; Apply equivalent subjects<br>Search modes - Boolean/Phrase	Interface - EBSCOhost Research Databases<br>Search Screen - Advanced Search<br>Database - APA PsycInfo	Display
S50	DE "Middle East Respiratory Syndrome"	Expanders - Apply related words; Apply equivalent subjects<br>Search modes - Boolean/Phrase	Interface - EBSCOhost Research Databases<br>Search Screen - Advanced Search<br>Database - APA PsycInfo	Display
S49	(DE "Severe Acute Respiratory Syndrome")	Expanders - Apply related words; Apply equivalent subjects<br>Search modes - Boolean/Phrase	Interface - EBSCOhost Research Databases<br>Search Screen - Advanced Search<br>Database - APA PsycInfo	Display
S48	DE "Pandemics"	Expanders - Apply related words; Apply equivalent subjects<br>Search modes - Boolean/Phrase	Interface - EBSCOhost Research Databases<br>Search Screen - Advanced Search<br>Database - APA PsycInfo	Display
S47	DE "Disease Outbreaks" OR DE "Epidemics"	Expanders - Apply related words; Apply equivalent subjects<br>Search modes - Boolean/Phrase	Interface - EBSCOhost Research Databases<br>Search Screen - Advanced Search<br>Database - APA PsycInfo	Display
S46	DE "Refugees"	Expanders - Apply related words; Apply equivalent subjects<br>Search modes - Boolean/Phrase	Interface - EBSCOhost Research Databases<br>Search Screen - Advanced Search<br>Database - APA PsycInfo	Display
S45	DE "War"	Expanders - Apply related words; Apply equivalent subjects<br>Search modes - Boolean/Phrase	Interface - EBSCOhost Research Databases<br>Search Screen - Advanced Search<br>Database - APA PsycInfo	Display

S44	DE "Conflict" OR DE "Violence" OR DE "War"	Expanders - Apply related words; Apply equivalent subjects<br>Search modes - Boolean/Phrase	Interface - EBSCOhost Research Databases<br>Search Screen - Advanced Search<br>Database - APA PsycInfo	Display
S43	DE "Disasters" OR DE "Natural Disasters"	Expanders - Apply related words; Apply equivalent subjects<br>Search modes - Boolean/Phrase	Interface - EBSCOhost Research Databases<br>Search Screen - Advanced Search<br>Database - APA PsycInfo	Display
S42	"collective N3 efficacy"	Expanders - Apply related words; Apply equivalent subjects<br>Search modes - Boolean/Phrase	Interface - EBSCOhost Research Databases<br>Search Screen - Advanced Search<br>Database - APA PsycInfo	Display
S41	"social support*"	Expanders - Apply related words; Apply equivalent subjects<br>Search modes - Boolean/Phrase	Interface - EBSCOhost Research Databases<br>Search Screen - Advanced Search<br>Database - APA PsycInfo	84,205
S40	"social network*"	Expanders - Apply related words; Apply equivalent subjects<br>Search modes - Boolean/Phrase	Interface - EBSCOhost Research Databases<br>Search Screen - Advanced Search<br>Database - APA PsycInfo	38,016
S39	"social norm*"	Expanders - Apply related words; Apply equivalent subjects<br>Search modes - Boolean/Phrase	Interface - EBSCOhost Research Databases<br>Search Screen - Advanced Search<br>Database - APA PsycInfo	13,733
S38	"social cohesion"	Expanders - Apply related words; Apply equivalent subjects<br>Search modes - Boolean/Phrase	Interface - EBSCOhost Research Databases<br>Search Screen - Advanced Search<br>Database - APA PsycInfo	Display
S37	"social capital"	Expanders - Apply related words; Apply equivalent subjects<br>Search modes - Boolean/Phrase	Interface - EBSCOhost Research Databases<br>Search Screen - Advanced Search<br>Database - APA PsycInfo	Display
S36	DE "Social Support"	Expanders - Apply related	Interface - EBSCOhost	Display

		words; Apply equivalent subjects<br>Search modes - Boolean/Phrase	Research Databases<br>Search Screen - Advanced Search<br>Database - APA PsycInfo	
S35	DE "Social Networks"	Expanders - Apply related words; Apply equivalent subjects<br>Search modes - Boolean/Phrase	Interface - EBSCOhost Research Databases<br>Search Screen - Advanced Search<br>Database - APA PsycInfo	Display
S34	DE "Social Norms"	Expanders - Apply related words; Apply equivalent subjects<br>Search modes - Boolean/Phrase	Interface - EBSCOhost Research Databases<br>Search Screen - Advanced Search<br>Database - APA PsycInfo	Display
S33	DE "Group Cohesion"	Expanders - Apply related words; Apply equivalent subjects<br>Search modes - Boolean/Phrase	Interface - EBSCOhost Research Databases<br>Search Screen - Advanced Search<br>Database - APA PsycInfo	Display
S32	DE "Social Capital"	Expanders - Apply related words; Apply equivalent subjects<br>Search modes - Boolean/Phrase	Interface - EBSCOhost Research Databases<br>Search Screen - Advanced Search<br>Database - APA PsycInfo	Display
S31	"women's health"	Expanders - Apply related words; Apply equivalent subjects<br>Search modes - Boolean/Phrase	Interface - EBSCOhost Research Databases<br>Search Screen - Advanced Search<br>Database - APA PsycInfo	Display
S30	"reproductive rights"	Expanders - Apply related words; Apply equivalent subjects<br>Search modes - Boolean/Phrase	Interface - EBSCOhost Research Databases<br>Search Screen - Advanced Search<br>Database - APA PsycInfo	Display
S29	("gender-based violence" OR "spouse abuse" OR "intimate partner violence" OR DE "Intimate Partner Violence" OR DE "Sexual Abuse")	Expanders - Apply related words; Apply equivalent subjects<br>Search modes - Boolean/Phrase	Interface - EBSCOhost Research Databases<br>Search Screen - Advanced Search<br>Database - APA PsycInfo	Display
S28	"maternal health"	Expanders - Apply related words; Apply equivalent	Interface - EBSCOhost Research Databases	Display

		subjects<br>Search modes - Boolean/Phrase	Search Screen - Advanced Search<br>Database - APA PsycInfo	
S27	"maternal and newborn health"	Expanders - Apply related words; Apply equivalent subjects<br>Search modes - Boolean/Phrase	Interface - EBSCOhost Research Databases<br>Search Screen - Advanced Search<br>Database - APA PsycInfo	Display
S26	STIs	Expanders - Apply related words; Apply equivalent subjects<br>Search modes - Boolean/Phrase	Interface - EBSCOhost Research Databases<br>Search Screen - Advanced Search<br>Database - APA PsycInfo	Display
S25	AIDS	Expanders - Apply related words; Apply equivalent subjects<br>Search modes - Boolean/Phrase	Interface - EBSCOhost Research Databases<br>Search Screen - Advanced Search<br>Database - APA PsycInfo	Display
S24	HIV	Expanders - Apply related words; Apply equivalent subjects<br>Search modes - Boolean/Phrase	Interface - EBSCOhost Research Databases<br>Search Screen - Advanced Search<br>Database - APA PsycInfo	Display
S23	"HIV/AIDS"	Expanders - Apply related words; Apply equivalent subjects<br>Search modes - Boolean/Phrase	Interface - EBSCOhost Research Databases<br>Search Screen - Advanced Search<br>Database - APA PsycInfo	Display
S22	("prenatal healthcare" OR "prenatal care" OR "postnatal care" OR "postnatal healthcare" OR "perinatal care" OR perinatal healthcare" OR "antenatal care" OR "antenatal healthcare")	Expanders - Apply related words; Apply equivalent subjects<br>Search modes - Boolean/Phrase	Interface - EBSCOhost Research Databases<br>Search Screen - Advanced Search<br>Database - APA PsycInfo	Display
S21	abortion	Expanders - Apply related words; Apply equivalent subjects<br>Search modes - Boolean/Phrase	Interface - EBSCOhost Research Databases<br>Search Screen - Advanced Search<br>Database - APA PsycInfo	Display
S20	contracept*	Expanders - Apply related	Interface - EBSCOhost	Display

		words; Apply equivalent subjects<br>Search modes - Boolean/Phrase	Research Databases<br>Search Screen - Advanced Search<br>Database - APA PsycInfo	
S19	"family planning"	Expanders - Apply related words; Apply equivalent subjects<br>Search modes - Boolean/Phrase	Interface - EBSCOhost Research Databases<br>Search Screen - Advanced Search<br>Database - APA PsycInfo	Display
S18	pregnan*	Expanders - Apply related words; Apply equivalent subjects<br>Search modes - Boolean/Phrase	Interface - EBSCOhost Research Databases<br>Search Screen - Advanced Search<br>Database - APA PsycInfo	Display
S17	"reproductive health"	Expanders - Apply related words; Apply equivalent subjects<br>Search modes - Boolean/Phrase	Interface - EBSCOhost Research Databases<br>Search Screen - Advanced Search<br>Database - APA PsycInfo	Display
S16	"sexual health"	Expanders - Apply related words; Apply equivalent subjects<br>Search modes - Boolean/Phrase	Interface - EBSCOhost Research Databases<br>Search Screen - Advanced Search<br>Database - APA PsycInfo	Display
S15	"sexual and reproductive health"	Expanders - Apply related words; Apply equivalent subjects<br>Search modes - Boolean/Phrase	Interface - EBSCOhost Research Databases<br>Search Screen - Advanced Search<br>Database - APA PsycInfo	Display
S14	DE "Adolescent Pregnancy"	Expanders - Apply related words; Apply equivalent subjects<br>Search modes - Boolean/Phrase	Interface - EBSCOhost Research Databases<br>Search Screen - Advanced Search<br>Database - APA PsycInfo	Display
S13	DE "Sexually Transmitted Diseases" OR DE "AIDS" OR DE "Gonorrhea" OR DE "Herpes Genitalis" OR DE "HIV" OR DE "Syphilis"	Expanders - Apply related words; Apply equivalent subjects<br>Search modes - Boolean/Phrase	Interface - EBSCOhost Research Databases<br>Search Screen - Advanced Search<br>Database - APA PsycInfo	Display
S12	DE "AIDS (Attitudes Toward)"	Expanders - Apply related words; Apply equivalent	Interface - EBSCOhost Research Databases	Display

		subjects<br>Search modes - Boolean/Phrase	Search Screen - Advanced Search<br>Database - APA PsycInfo	
S11	DE "AIDS"	Expanders - Apply related words; Apply equivalent subjects<br>Search modes - Boolean/Phrase	Interface - EBSCOhost Research Databases<br>Search Screen - Advanced Search<br>Database - APA PsycInfo	Display
S10	DE "HIV" OR DE "AIDS"	Expanders - Apply related words; Apply equivalent subjects<br>Search modes - Boolean/Phrase	Interface - EBSCOhost Research Databases<br>Search Screen - Advanced Search<br>Database - APA PsycInfo	Display
S9	(DE "Prenatal Care" OR DE "Postnatal Period" OR DE "Perinatal Period")	Expanders - Apply related words; Apply equivalent subjects<br>Search modes - Boolean/Phrase	Interface - EBSCOhost Research Databases<br>Search Screen - Advanced Search<br>Database - APA PsycInfo	Display
S8	DE "Abortion (Attitudes Toward)"	Expanders - Apply related words; Apply equivalent subjects<br>Search modes - Boolean/Phrase	Interface - EBSCOhost Research Databases<br>Search Screen - Advanced Search<br>Database - APA PsycInfo	Display
S7	DE "Induced Abortion"	Expanders - Apply related words; Apply equivalent subjects<br>Search modes - Boolean/Phrase	Interface - EBSCOhost Research Databases<br>Search Screen - Advanced Search<br>Database - APA PsycInfo	Display
S6	DE "Birth Control" OR DE "Contraceptive Devices" OR DE "Rhythm Method" OR DE "Sterilization (Sex)"	Expanders - Apply related words; Apply equivalent subjects<br>Search modes - Boolean/Phrase	Interface - EBSCOhost Research Databases<br>Search Screen - Advanced Search<br>Database - APA PsycInfo	Display
S5	DE "Family Planning Attitudes"	Expanders - Apply related words; Apply equivalent subjects<br>Search modes - Boolean/Phrase	Interface - EBSCOhost Research Databases<br>Search Screen - Advanced Search<br>Database - APA PsycInfo	Display
S4	DE "Family Planning" OR DE "Birth Control" OR DE "Delayed Parenthood"	Expanders - Apply related words; Apply equivalent subjects	Interface - EBSCOhost Research Databases<br>Search Screen - Advanced	Display

		Search modes - Boolean/Phrase	Search<br>Database - APA PsycInfo	
S3	(DE "Pregnancy") OR (DE "Pregnancy" OR DE "Adolescent Pregnancy" OR DE "Pregnancy Outcomes" OR DE "Primipara")	Expanders - Apply related words; Apply equivalent subjects<br>Search modes - Boolean/Phrase	Interface - EBSCOhost Research Databases<br>Search Screen - Advanced Search<br>Database - APA PsycInfo	Display
S2	DE "Reproductive Health"	Expanders - Apply related words; Apply equivalent subjects<br>Search modes - Boolean/Phrase	Interface - EBSCOhost Research Databases<br>Search Screen - Advanced Search<br>Database - APA PsycInfo	Display
S1	DE "Sexual Health"	Expanders - Apply related words; Apply equivalent subjects<br>Search modes - Boolean/Phrase	Interface - EBSCOhost Research Databases<br>Search Screen - Advanced Search<br>Database - APA PsycInfo	Display
