# Supplementary figures and images for: The role of social capital in women’s sexual and reproductive health and rights in humanitarian settings: a systematic review of qualitative studies
Source: Confl Health. 2021 Nov 24;15:87. doi: 10.1186/s13031-021-00421-1 (PMC8611620; doi:10.1186/s13031-021-00421-1)

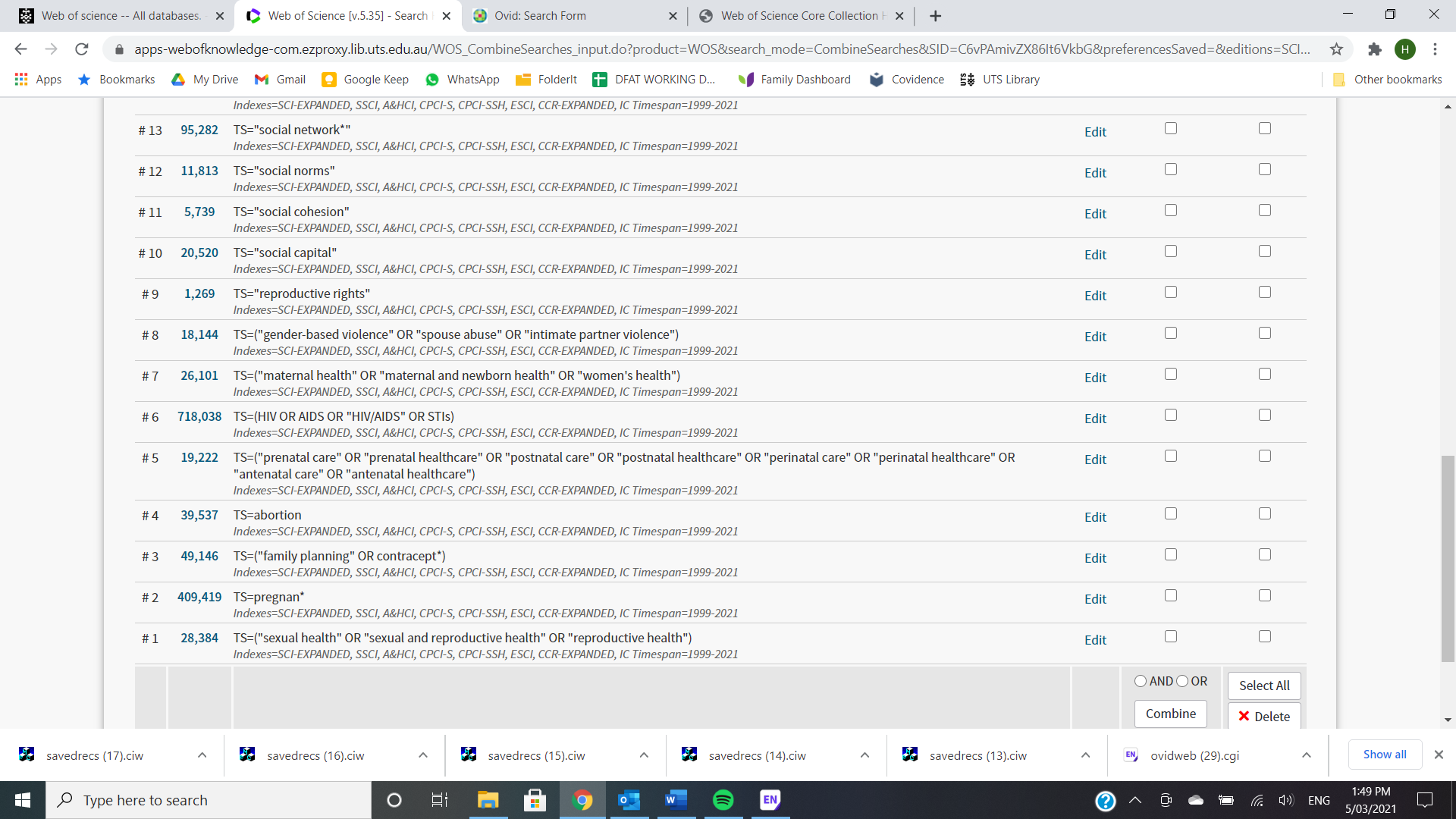


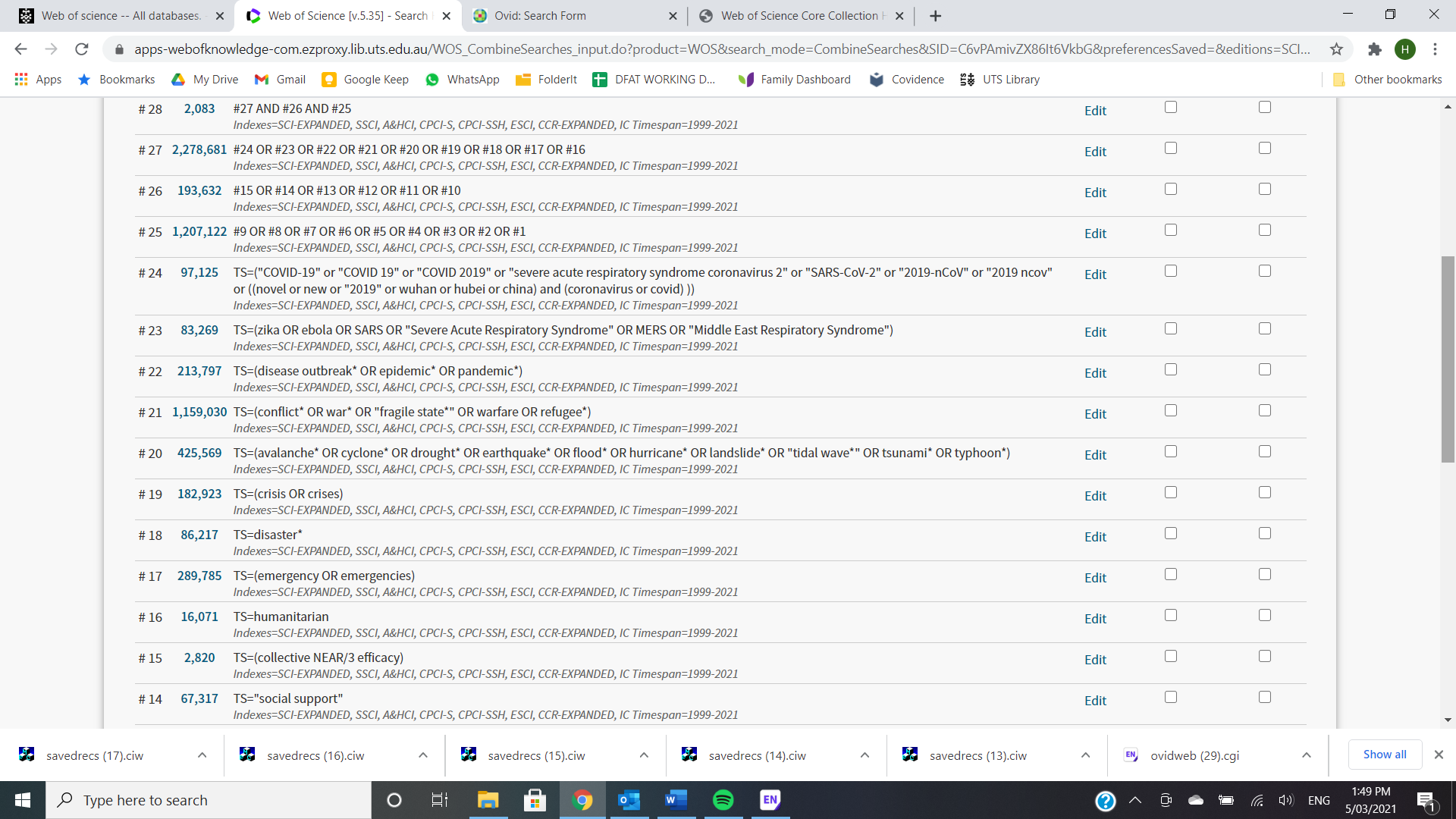

Supplement: Supplementary file 6 — Additional file 6. Web of Science Search Strategy. [file 13031_2021_421_MOESM6_ESM.docx]
